# Utility of Antibodies in the Diagnoses of Thyroid Diseases: A Review Article

**DOI:** 10.7759/cureus.31233

**Published:** 2022-11-08

**Authors:** Amit K Gupta, Sunil Kumar

**Affiliations:** 1 Medicine, Jawaharlal Nehru Medical College, Datta Meghe Institute of Medical Sciences, Wardha, IND

**Keywords:** autoimmune thyroid illnesses, graves' disease in pregnancy, hypothyroidism, hyperthyroidism, graves' disease, antithyroid antibodies, trab clinical usefulness, trab diagnostic usage

## Abstract

Thyroid problems are among the most widespread endocrine illnesses, affecting individuals in India and the global population. A thyroid function test is used to diagnose, screen, and monitor patients. Hyperthyroidism is a clinical condition due to excessive circulation of thyroid hormone; in contrast, hypothyroidism is due to a deficiency of thyroid hormone. Graves' disease (GD) is a form of hyperthyroidism due to thyroid-stimulating hormone receptor autoantibodies (TRAb), and anti-thyroid peroxidase antibodies (anti-TPO antibodies). The most common reason for hypothyroidism is Hashimoto's thyroiditis (HT), in which patients have thyroid receptor antibodies (TRAb), antibodies to thyroid peroxidase (TPO), and thyroglobulin antibodies. Many essential genes, including the thyroid-specific genes thyroglobulin (TSGT), TSH-receptor gene, human leukocyte antigen (HLA) genes, cytotoxic T lymphocyte-associated antigen (CTLA) genes, thyroglobulin gene, vitamin D receptor gene, and many immune-regulatory genes were associated with autoimmune thyroid diseases' (AITDs') etiology. This review paper aims to determine if antibodies are beneficial in detecting autoimmune thyroid disease or not. We have also discussed the etiology of autoimmune thyroid illness, serum antibodies in autoimmune thyroid disease, pathophysiology, and TSH receptor features.

## Introduction and background

**Figure 1:**
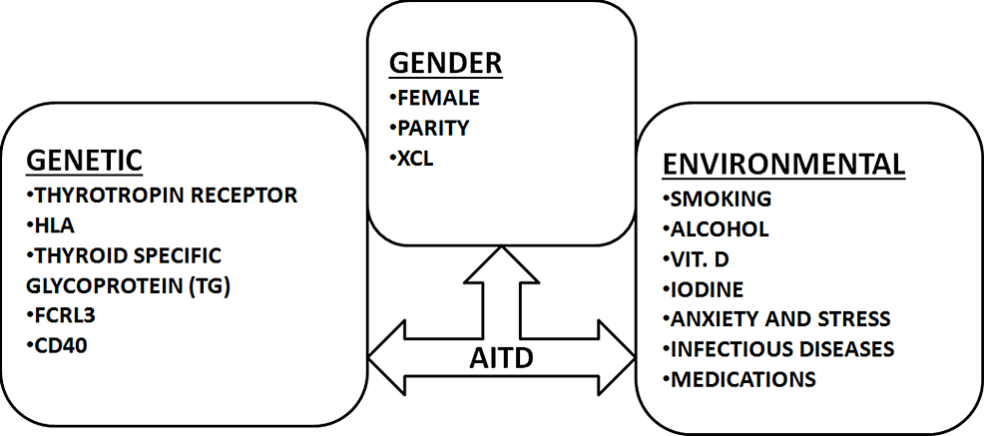
Etiopathogenesis of autoimmune thyroid disease, multifactorial HLA: Human leukocyte antigens; FCRL: Fc Receptor-like; CD40: Cluster Of Differentiation 40; XCL: Xerocomus Chrysenteron Lectin; VIT. D: Vitamin D; AITD: Autoimmune thyroid disease Source [1-5]

Recent epidemiological research found that the prevalence of several autoimmune endocrine illnesses, such as autoimmune thyroid disease (AITD), has been steadily rising [1]. The complex etiology of AITD includes genetic and environmental factors; females are more likely to be affected, as shown in Figure 1. Graves' disease (GD) and Hashimoto's (HT), which make up the majority of cases of AITD, have a high correlation in those over the age of 45 to 50 years. These patients have high levels of autoantibodies against thyroid proteins, namely thyroglobulin, thyroid peroxidase, and thyroid stimulating hormone receptors antibodies (TRAb). Genes such as the truncated short GalTase (TSGT) protein and thyroid stimulating hormone (TSH) receptor, as well as many immune-regulatory genes, were also found in association with AITD [1,2]. AITD has a complex etiology due to autoimmunity against thyroid-antigen (Ag). Genetic and environmental factors play an important role in AITD etiology. Although AITD is an archetypal organ-specific autoimmune illness, it is unclear what causes these autoimmune reactions [3].

## Review

Methodology

**Figure 2:**
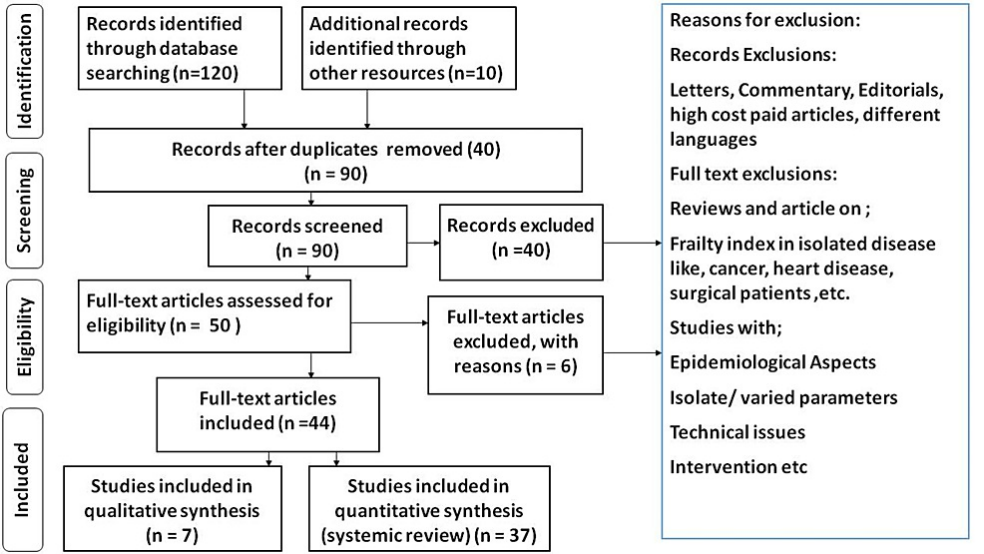
PRISMA model for the search strategy Importance of antibodies in thyroid disease and their role in its diagnosis; studies devoted entirely to the antibodies in thyroid disease were included.

Google, PubMed, Medline, Embase, and other electronic databases were used to search the English-language literature. Anti-thyroid antibodies, Graves' disease (GD), hyperthyroidism, hypothyroidism, pregnancy-related GD, and autoimmune thyroid diseases were the search phrases. They also included TSH Receptor Antibodies (TRAb) diagnostic and clinical usefulness. The authors' expertise and experience in the subject area aided the preservation of pertinent publications. The articles in this review meet the following requirements: There are studies in English; There are studies specifically focused on TRAb, GD, and Thyroid stimulating hormone receptors. The PRISMA research approach is displayed in Figure 2.

Serum antibodies in autoimmune thyroid diseases (AITD)

Thyroid Peroxidase Autoantibody

It is one of the first thyroid antibodies identified. It was later discovered that this antibody targets Thyroid Peroxidase (TPO), and these antibodies are used to diagnose AITD. The production of thyroid hormones is carried out by the trans-membrane protein TPO, which is found in the apical membrane of thyrocytes, and antibodies to TPO lead to AITD [1,4,5].

TRAb

Anti-TSHR antibodies are classified as stimulating, blocking, and neutral types based on the ability to connect with various kinds of epitopes, i.e., linear and conformational, and the diversity of biological activities they perform. TSHR-stimulating antibodies cause GD; however, their function in persons with Hashimoto's thyroiditis (HT) is contested [6,7].

Thyroglobulin Specific Antibodies (TgAb)

More than 90% of those with HT have TgAb, which is also detectable in small amounts in the serum of those with GD [8]. Twenty percent of people with normal thyroid function in the general population also have TgAb, which is most likely a sign of a sub-clinical AITD [1].

Pendrin Antibodies

Iodine transport is aided by pendrin, and AITD has been linked to sequence variations in the pendrin gene. Anti-pendrin antibodies were present in 74% of those with GD and about 97% of people with HT [9,10].

Characteristics of TSH receptor 

The TSH-Receptor (TSHR) is a G-protein-coupled transmembrane receptor. The gene is on the long arm of chromosome 14q31 [11-13]. There are several ways (like Synthetic peptide sequences, anti-peptide antibodies, and site-directed mutagenesis) to map the regions of the TSHR where TSH or TRAb bind, but each has substantial drawbacks [11,14]. TRAb have a significant influence on the pathophysiology of GD-induced hyperthyroidism. Multiple assays to identify TRAb indicate at least two types; the first class can boost thyroid adenylate cyclase activity, while the second class can prevent TSH from binding with its receptor. These autoantibodies are detected in 50-100% of untreated GD patients and are altered by administering anti-thyroid medications (ATD), surgery, or I^131^. TSH antibodies are also linked to thyroid illnesses other than GD, such as HT, toxic adenoma, subacute thyroiditis, and myxoedema [15].

TRAb and the use of TRAb tests

Actual GD cannot exist in the absence of TRAb. TRAb testing should thus be highly beneficial in identifying GD, but TRAb testing is not used initially to determine the etiology of hyperthyroidism, despite this obvious fact. The American Thyroid Association (ATA) and the American Association of Clinical Endocrinologists propose a thyroid scan as the primary diagnostic method in their joint guidelines [16,17]. The British Thyroid Association advises testing for TRAb except in exceptional circumstances [16]. Radio-iodine uptake and scanning (RAIU+S) continue to give a reliable evaluation of thyroid disorders. While deciding how much I^131^ to administer to patients for therapy, many doctors employ RAIU+S. RAIU+S must be avoided in lactating mothers. Aside from economic considerations, RAIU+S is still the gold standard [16]. To differentiate between GD and subacute painless thyroiditis (SPT), TRAb testing is helpful. SPT clinical presentation may include thyrotoxicosis and diffuse non-nodular goiter. Still, thyrotoxicosis in SPT is self-limited and recovers spontaneously after a few weeks due to autoimmune damaging events rather than continuous hormone production. Thyroid peroxidase antibodies have been found in almost all SPT patients and up to 70% of GD patients [18]. 

TRAb testing is beneficial in differentiating postpartum thyroiditis from recurrent GD. A particular worry is that individuals with SPT or postpartum thyroiditis might have a positive TRAb. These individuals most likely have hyperthyroidism [16,19,20]. Because of these factors, a TRAb test that is more cost-effective than RAIU+S is advised as the first diagnostic strategy for overt hyperthyroidism to identify individuals with GD and reduce the need for additional testing. In uncertain cases, determining the T3/T4 ratio may be used. RAIU+S, which is expensive, would only be provided to those whose thyrotoxicosis was not caused by GD, as evidenced by a negative TRAb, or to people for whom radio-iodine was determined to be the appropriate course of treatment. One downside of this method is that the clinical performance of TRAb in persons with subclinical hyperthyroidism has not been well investigated [21]. In one study, four of the eleven elderly participants who exhibited diffuse uptake on a thyroid scan and subclinical hyperthyroidism also had negative TRAb findings [22,23]. GD has flare-ups and remissions like many other autoimmune disorders. A drug cessation study was done to establish if remission was achieved in individuals treated with antithyroid medication. Around 50% of individuals who have not attained immunological remission are subject to the dangers of recurrent hyperthyroidism [5,24,25]. Due to the low sensitivity, older TRAb tests cannot provide substantial prediction value. The finest example of this may be found in a meta-analysis, Feldt-Rasmussen et al., in which only 53% of relapsing patients had positive TRAb results, compared to 39% of negative patients [24,26].

Most studies labelled individuals as relapsing if their hyperthyroidism reappeared three years after stopping antithyroid medications. No matter how accurate the assay is, it is assumed that the absence of TRAb in a patient's history cannot guarantee that it will not recur in the far future. The research by Massart et al. compared the Trafficking Kinesin Protein (TRAK) test after an 18-month methimazole treatment with two different M22-based assays. The enzyme-linked immunosorbent assay (ELISA) test detected 21 of 62 patients who relapsed up to three years later and were TRAb-negative, which has a negative predictive value of 64.4%. One must consider whether it is reasonable to expect a straightforward serological test to predict reliably whether the patient would be free of illness over the next three years, given the unpredictable nature of the autoimmune response. The study addressed the prediction of relapses and showed a relapse in the first six months after treatment discontinuation [27,28]. Depending on the situation, patients with negative or low-titer TRAb (in remission) may stop using methimazole. Patients with a high risk of complications, such as those with paroxysmal atrial fibrillation, may benefit most from RAI therapy or continued antithyroid drug treatment because sustained remission cannot be predicted based on a current negative TRAb level. Patients with medium- to high-titer of TRAb should be given the option of either definitive treatment or continuing methimazole therapy and repeat testing every six months to a year. They should be aware that stopping treatment would probably result in a rapid recurrence [22-28].

Pregnancy and TRAb

Like other Immunoglobulin G (IgG), TRAb may easily cross the placenta during pregnancy in GD patients. As a result, they can activate the fetal thyroid gland, leading to fetal thyrotoxicosis [29]. Because of this, pregnant women with GD who have high levels of TRAb are considered high-risk pregnancies. Fetal thyrotoxicosis can have significant side effects on the mother and baby, like intrauterine death, growth retardation, heart failure, fetal hydrops, placental abruption, premature birth, etc., if it is not treated [30]. Pregnancy is an immunosuppressive situation, and because TRAb levels often fall, this problem is partially relieved. Using first-generation radio iodine assay (RIA), a study of 45 GD women revealed a substantial drop in TRAb levels [31]. A more recent Japanese study showed a significant drop in TRAb levels between early and late pregnancy. In general, fetal and neonatal thyrotoxicosis caused by maternal GD is a widespread issue [32-35]. The presence of TRAb in pregnant women with GD is the best indicator of fetal and neonatal hyperthyroidism, which has been shown to have a predictive value of 42%. So, according to the most recent ATA standards, TRAb should be measured between 24 and 28 weeks of gestation, and the fetus should be actively observed if the value of TRAb is more significant than three times the standard upper limit [36-38]. In one significant research, only nine of 788 neonates (out of 1.6 million infants tested from 1984 to 1989) in whom New York State Newborn screening Tests were performed revealed congenital hypothyroidism had positive TRAb [5,39-42].

The following are the European Thyroid Association's recommendations for testing TRAb during pregnancy: There is no need to try for TRAb in euthyroid women who are untreated or have recently undergone antithyroid medication. In women who have had Graves' disease surgery or radioiodine treatment, TRAb is assessed early in pregnancy. If the level is elevated, the mother should be treated with antithyroid medications, and the fetus should be watched for hyperthyroidism symptoms. Titers of antibodies should be rechecked in the third trimester. Women on antithyroid medication must change their medicines for the mother's free T4 level to return to normal. The optimum time to look for TRAb is in the third trimester [11,43].

TRAb and Graves' ophthalmopathy (GO)

Although the involvement of TRAb in the etiology of GO is yet unclear, the incidence and severity of GO in hyperthyroid patients rise with TRAb concentration. More than 90% of patients in an experiment on euthyroid individuals with GO had positive TRAb [43,44]. Limited information is available on the use of TRAb for predicting and tracking GO responses to treatment. In conclusion, the evidence currently available justify the regular use of the TRAb test in patients with euthyroid GO to confirm the diagnosis. It is anticipated that adopting more accurate assays will provide a better understanding of TRAb in GO.

## Conclusions

Our ability to accurately test for antibodies in patients with thyroid disorders has dramatically improved due to recent technological advancements. TRAb in patients on methimazole help in predicting the outcome of the treatment. It is also used to indicate the neonatal transmission of GD. The main goal is to reassure the majority of GD-positive women who had negative or low titers of antibodies. The connection between thyroid and ophthalmopathy in GD continues to become apparent. Utilizing the most recent assays, antibodies (thyroid peroxidase autoantibodies, TRAb, thyroglobulin specific antibodies) are proving to be a potent marker (if not pathogen) of thyroid disorders, and the subsequent months are expected to provide further insight into its function in this state.
